# Effects of polymerase, template dilution and cycle number on PCR based 16 S rRNA diversity analysis using the deep sequencing method

**DOI:** 10.1186/1471-2180-10-255

**Published:** 2010-10-12

**Authors:** Jin-Ya Wu, Xiao-Tao Jiang, Yun-Xia Jiang, Su-Ying Lu, Fei Zou, Hong-Wei Zhou

**Affiliations:** 1 Department of Environmental Health, School of Public Health and Tropical Medicine, Southern Medical University, Guangzhou, Guangdong, 510515, China

## Abstract

**Background:**

The primer and amplicon length have been found to affect PCR based estimates of microbial diversity by pyrosequencing, while other PCR conditions have not been addressed using any deep sequencing method. The present study determined the effects of polymerase, template dilution and PCR cycle number using the Solexa platform.

**Results:**

The *PfuUltra II *Fusion HS DNA Polymerase (Stratagene) with higher fidelity showed lower amount of PCR artifacts and determined lower taxa richness than the *Ex Taq *(Takara). More importantly, the two polymerases showed different efficiencies for amplifying some of very abundant sequences, and determined significantly different community structures. As expected, the dilution of the DNA template resulted in a reduced estimation of taxa richness, particularly at the 200 fold dilution level, but the community structures were similar for all dilution levels. The 30 cycle group increased the PCR artifacts while comparing to the 25 cycle group, but the determined taxa richness was lower than that of the 25 cycle group. The PCR cycle number did not changed the microbial community structure significantly.

**Conclusions:**

These results highlight the PCR conditions, particularly the polymerase, have significant effect on the analysis of microbial diversity with next generation sequencing methods.

## Background

Microbial diversity in sediment or soil environments is very high, but the exact number of the taxa richness remains elusive [1]. The estimated bacterial species ranged from nearly 10^3 ^[2] to over 10^6 ^[3] in a gram of sediment sample. Nevertheless, the figure has never been verified because of the low throughput of the traditional 16 S rRNA clone library method. Determining 16 S rRNA short variable tags using the pyrosequencing provided an unprecedented sequencing depth with tens to hundreds of thousands of tags per sample [4,5], and the method regenerated people's interest in measuring and comparing the microbial taxa richness in various samples [6-8]. Nevertheless, two major types of problems about the 16 S rRNA pyrosequencing process were shortly revealed.

One was that, in any determined samples, the rarefaction curve, particularly for the unique operational taxonomy units (OTU) (100% similarity), never approached asymptotic. The highest number of sequences for a single sample (442,058) was performed on a deep marine biosphere, but the rarefaction curve of the 0.03 distance OTU (97% similarity) was still increasing steeply [4]. The ever-increasing number of different tags either reflects a real microbial taxa richness being detectable only with a higher sequencing effort, or they are artifacts produced by PCR or sequencing processes. Recently, Quince et al. (2009) found that the base calling error of the pyrosequencing method significantly increased the number of novel unique sequences. Consequently, the escalating number of the unique tag, particularly the singletons (tags occur only once) [9], might be produced mainly from experimental artifacts of pyrosequencing, rather than from the true diversity; and the pyrosequencing method was suggested to overestimate the taxa richness accordingly [10,11].

The other type of problems was that the microbial diversity might be skewed by experimental procedures, particularly by PCR. Studies suggested that the PCR primer and amplicon length affected the estimation of species richness and evenness [12,13], and the primers missed half of rRNA microbial diversity [1]. In addition to primers, the effect of some other PCR conditions, like PCR cycle number, annealing temperature et al., have been evaluated with the traditional 16 S rRNA clone library or fingerprinting methods [9,14-16], but their effects have never been assessed with any next generation sequencing approach yet.

Very recently, we developed a barcoded Illumina paired end sequencing (BIPES) method to determine the 16 S rRNA V6 tags by pair end sequencing strategy on another next generation sequencing platform, the Illumina systems [17]. In the present study, we report our evaluation of three PCR conditions, namely template dilution, PCR cycle number and polymerase, on the V6 microbial diversity analysis.

## Results

### Deep sequencing result

A total of 10 samples for 5 PCR conditions, each in replicate, were determined. All samples were amplified using the same tube of DNA template (34 ng μl^-1^) extracted from a sediment sample collected at the edge of a mangrove forest. The V6 fragment of each sample was amplified with a different barcoded upstream primer and all PCR products were pooled together and sequenced. We determined 75 bases from both end of the PCR amplicons (paired-end sequencing) on a Solexa GAII platform. After sequencing, each read was cut to 60 base length from the 5' end because the sequencing error increased significantly after the site. The pair end reads were overlapped, with at least 5 bp connected, to construct the full length sequences of the V6 amplicons. We only collected high quality sequences with 0 mismatches in the overlapped region for further diversity analysis, and 605,605 tags were obtained. To minimize potential sequencing errors, we further trimmed sequences with a stringent condition, which was removing all tags with any mismatches within primers (52,016), with any N bases (23,222) or less than 35 bp for the V6 regions (484). Finally, we obtained 529,883 clean and high quality sequences for the 10 samples and they were allocated to specific samples according to barcode sequences (Table 1).

**Table 1:** Sample list

ID	Barcode	PCR conditions			Read number		Chao1		Ace	
		T*	**C**^&^	**E^$^**	**(total)**	**(unique)**	**(unique)**	**0.03**	**(unique)**	**0.03**

A1	TGGAGTAG	1	30	Ex	83,194	17,841	58,148	13,020	108,316	18,590
A2	TGTGACTG	1	30	Ex	158,519	30,361	55,899	34,096	107,984	22,871
B1	CAGACAGA	20	30	Ex	52,793	12,874	39,159	7,455	69,614	9,274
B2	CAGTGAGA	20	30	Ex	78,392	16,846	50,838	8,986	88,268	10,782
C1	CATCTCGT	200	30	Ex	25,705	6,013	16,586	2,700	24,554	2,669
C2	GGTAGGAT	200	30	Ex	25,514	5,968	16,828	2,731	25,294	2,649
D1	GTGTAGAG	20	25	Ex	10,833	3,992	13,749	4,457	26,155	6,406
D2	GTTGGTAC	20	25	Ex	25,181	7,578	22,921	6,698	42,784	9,517
E1	GTCAGAGA	20	30	Pfu	34,600	6,750	17,853	6,332	30,589	9,255
E2	GTCTTCTG	20	30	Pfu	35,152	6,818	18,281	6,416	30,434	8,792
	Total				529,883	67,826	229,287	34,883	120,750	50,579

*: Dilution folds of the DNA template; &: PCR cycle number; $: Polymerase used (Ex, *Ex Taq *from Takara; Pfu, *PfuUltra II *Hotstart 2× Master Mix from Stratagene).

### Rarefaction analysis

**Figure 1:**
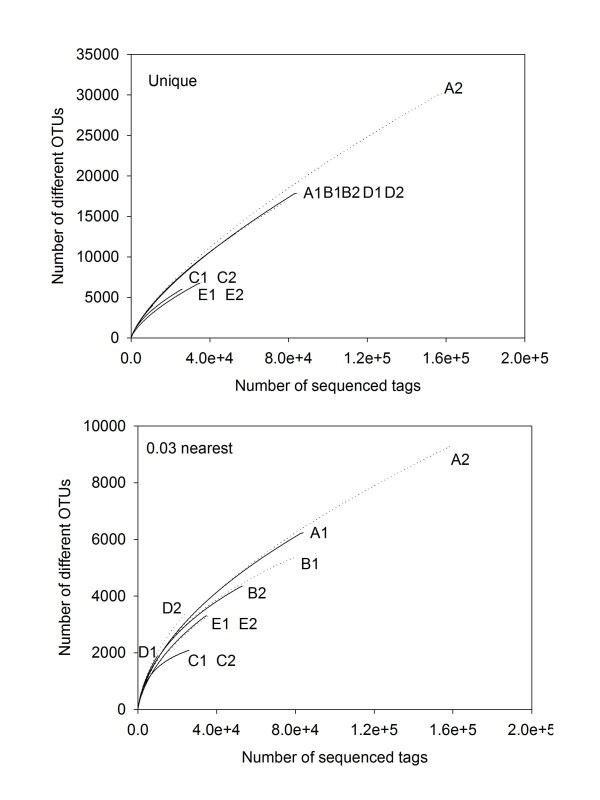
**Rarefaction curves for the 10 samples using 5 different PCR conditions**. A shows the unique (100% similarity) OTU. B shows 0.03 OTUs at a 97% similarity using the nearest neighbor clustering method.

We presented the rarefaction curves for OTUs at both unique and 0.03 distances (Fig. 1). The unique OTU represents both true diversity and PCR artifacts as described above, while the 0.03 distance OTU may mitigate the effect of PCR mutation artifacts, because the mutation rate in a ~60 bp V6 tag is less than 1 bp (< 3%) [9]. In our present study, we used the nearest distance, rather than the furthest distance, for calculating the OTUs using the Mothur [18]. The reason was that rarefaction curves with different sequencing depth showed consistent trajectory using the nearest distance, but changed with the furthest distance (Additional file 1).

Rarefaction curves for PCR replicates showed consistent trajectories for both unique and 0.03 OTUs (Fig. 1), indicating that the PCR and sequencing steps had good reproducibility. The unique curves for A (1 fold diluted template, 30 cycles), B (20 fold diluted template, 30 cycles) and D (20 fold diluted template, 25 cycles) conditions almost overlapped (Fig. 1A), indicating a similar richness of unique V6 tags in above three conditions. The C condition (200 fold diluted template, 30 cycles) showed a lower slope than the above three, indicating that dilution of DNA template from 20 to 200 fold reduced the V6 diversity of the sample. The E condition showed the lowest slop, proving that the polymerase had an obvious effect, as all conditions except polymerase for group E were the same as that for group B.

The 0.03 OTU curves were different with that of the unique OTU (Fig. 1B). The most marked change happened to A, B and D groups, which three showed dissimilar slopes this time. The condition D showed the steepest slope, suggesting that more tags in the group having larger than 3% variance than the other two conditions. The difference between E and B curves for 0.03 OTU was less pronounced than that for the unique OTU, indicating that a proportion of different unique sequences between B and E groups were within 97% similarity, which could possibly be produced by the PCR mutation.

In addition to unique and 0.03 OTUs, we also compared OTUs at 0.05 and 0.10 distances (Additional file 2), and the trends were generally similar to that for 0.03 OTU. Nevertheless, because the larger distance OTUs harbored more varied sequences, the differences between the 5 groups were less obvious.

### Abundance of top 300 tags

**Figure 2:**
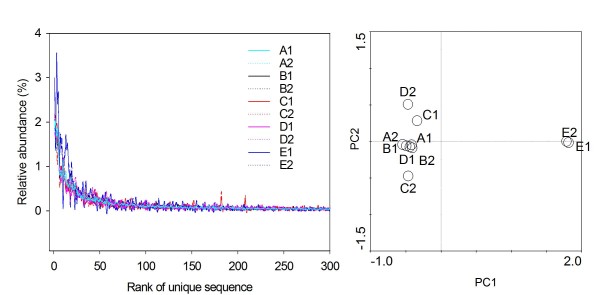
**Relative abundances (%) of the top 300 predominant V6 sequences in the 10 samples**. The right figure shows the PCA of the 10 samples using the abundance data of top 300 tags.

The Fig. 2 presents the relative abundance of the top 300 V6 sequences in the 10 samples. We observed that the E group (blue curve) showed significant differences with the other four groups, particularly for many tags within the top 50 abundances. For instance, the 10^th ^abundant tag assigned as *Syntrophobacterales *(*Deltaproteobacteria*) showed 0.95-1.19% abundance in A to D groups, but only occupied 0.03-0.06% in the E group. The 15^th ^abundant tag assigned as *Epsilonproteobacteria *had abundances of 0.46-0.62% in group A to D samples, but showed 1.50-1.53% in the E group. In total, 91 out of the top 300 tags in group E showed significant differences with other 8 samples using the students t-test analysis (p < 0.01). A further PCA analysis using the 300 tags proved that the E1 and E2 were obviously different with other 8 samples (Fig. 2).

### Microbial community structure

**Figure 3:**
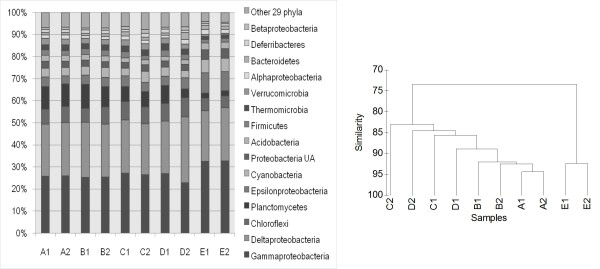
**Relative abundance of bacteria phyla (subphyla) in the 10 samples**. The dendrogram shows the clustering of 10 samples using the phyla (subphyla) abundance data.

The community structure was compared at the phylum (subphylum for proteobacteria) level (Fig. 3). In general, the A to D groups showed very similar structure, but the E group showed obvious differences. The A-D groups showed higher phylum evenness than the E group. Statistically, the E group had higher percentage of *Gammaproteobacteria *and *Epsilonproteobacteria*, but lower percentage of *Chloroflexi *and *Planctomycetes *(One Way ANOVA, p < 0.01). We also compared the 10 samples using clustering with Primer 6 (Fig. 3). The result showed that samples E1 and E2 formed a different branch with the other 8 samples.

## Discussion

### Sequencing quality

The present study sequenced the 16 S rRNA V6 tags using the Solexa platform, which employed a different base calling procedure with the pyrosequencing [19]. We do not assume that the Solexa platform have a higher sequencing accuracy than the 454. Nevertheless, as the sequencing accuracy of all next generation sequencing methods decreases at the 3' end of the reads [19], overlapping of the pair end sequencing reads with 5' end sequences obviously increases the accuracy of the final result. Furthermore, we employed a very stringent pipeline to trim the low quality reads, as we removed all tags with mismatches in the overlapped region, mismatches with primers, having any N bases, and very short tags. The large number of tags showing mismatches with primers (52,016) had two resources: (i) the impurity of the primers during primer synthesis; and (ii) sequencing error. We suggest that the first one could be the major reason as the quality checking of the primer using mass spectrum showed that there could be nearly 10% of impure primers in the ultra PAGE purified primers (Additional file 3). We found that removing tags with any N bases was very critical, as the 23,222 tags with N bases formed 16,397 unique sequences. Considering that the final number of unique tags was only 67,826, the tags with N bases could contribute a large number of novel unique sequences, but only as singletons or doubletons, therefore to increase the diversity estimation. Although we may not preclude the sequencing artifacts existing in the final result, we suppose that sequencing error effect has been minimized at the present time and we could explore the PCR effect on the 16 S rRNA deep sequencing methods.

### Effect of polymerase

The polymerase showed significant effect on both the taxa richness and community structure analysis in our result. Qiu et al. (2001) compared three enzymes with different processitivity and fidelity. They found that the Ampli*Taq *showed the lowest number PCR artifacts, but not the enzymes with higher fidelity or processitivity. In our study, the two tested polymerases were high fidelity enzymes. The *PfuUltra II *Fusion HS DNA Polymerase was suggested to have the highest fidelity (20 fold higher than the conventional *Taq*) and enhanced processitivity (Stratagene manual). The *Ex Taq *(Takara) had a 4 fold higher fidelity than the conventional *Taq*. The rarefaction curves of *PfuUltra *II at the unique distance showed much lower slopes than that of the *Ex Taq*, indicating that less PCR artifacts were produced using the *PfuUltra *II enzymes. In addition, while the determined sequences were grouped into 0.03 OTUs, the slopes of rarefaction curves of the two groups showed less pronounced differences, suggesting that a number of the different tags between the two groups could be PCR artifacts, as PCR mutants were suggested to be within 97% similarity with the original sequence [9].

A more important finding of the present study was that the two enzymes showed different community structures, besides different rRNA microbial richness. The data showed obviously that the two enzymes had significantly different efficiency for amplifying certain kinds of tags, even for the very abundant sequences. PCR bias was previously attributed to intrinsic differences in the amplification efficiency of templates [16] or to the primer binding energy and kinetics [9,20]. Our present study, for the first time, revealed the marked bias induced by different polymerase cocktails. It should be note that there were slight differences of Mg^2+ ^and dNTP concentrations between the two cocktails, but the major factor should be the polymerase. Arezi et al. (2003) found that polymerases showed different efficiencies while amplifying 5 templates varied in length or percentage GC content. The *pfu *enzyme showed higher efficiency to amplify long templates and high percentage GC content templates[21]. The different efficiently might be related to the processivity, in addition to the proof-reading function of the enzymes [22]. Although both enzymes used in our present study were high-fidelity enzymes, the *PfuUltra II *Fusion HS DNA Polymerase was suggested to have enhanced processivity; therefore the two enzymes might have different efficiencies for specific sequences. While amplifying the same 16 S rRNA mixture, we can assume that one enzyme might amplify diverse 16 S rRNA tags at similar efficiency, while the other one might be not, and the determined community structures would be different accordingly. We can deduce that the community structure at more specific taxonomic levels, e.g. genus or OTU, will change more obviously than the phylum level, as the abundant tags showed so large variances. Nevertheless, we cannot determine which one of the enzymes reflected the real microbial community structure currently, and studies using known 16 S rRNA amalgam as template are warranted.

### Effect of dilution

The present study for the first time explored the effect of template dilution on the microbial diversity analysis. It is well known that different soil or sediment DNA extraction methods yield different amount and purity of DNAs [23]. The residual humus and other contaminants in DNA may inhibit the PCR reaction and the DNA is usually diluted for PCR amplification by try and error. Nevertheless, if the dilution affects the diversity analysis has never been explored before. We discussed the template dilution fold rather than the absolute concentration, because 1 gram of different sediment samples might have very different amount of DNA, which should also be considered while analyzing the microbial diversity.

Dilution of the template obviously reduced the determined taxa richness, particularly from the 20 fold to 200 fold. The effect of dilution from 1 to 20 fold was less obvious than the above situation, indicating that the 1 fold DNA sample might be saturated and could endure a small fold of dilution. On the other hand, template dilution had few impacts on the microbial community structure determination, as the relative abundance of each unique OTU and the phylum structure showed good similarity among A, B and C groups. Therefore, previous studies using fingerprinting methods focusing on the structure of major OTUs should be consistent no matter how the template was diluted.

### Effect of cycle number

The effect of PCR cycle number has been determined before. More cycle numbers leads to accumulation of more point mutation artifacts [16] and people suggested to perform PCR at as few cycle numbers as possible [9,14]. In the present study, the 30 cycle and 25 cycle conditions showed similar rarefaction curves for the unique OTU, but the curves of the 0.03 OTU were different (Fig. 1). The data indicated that more unique OTUs in the 30 cycle group showed higher than 97% similarity, which might come from the PCR mutation, proving that more cycle numbers caused more point mutations. In addition, we found that less cycle number lead to a higher estimation of taxa richness even with fewer sequences (Table 1).

The cycle number did not show any significant effect on the community structure as some reports [9,14], which was different with the report that less cycle numbers increased the proportion of predominant groups [15]. It should be noted that the variation of replicate samples was slightly higher in the 25 cycle group, indicating that replicates or combining of different tubes should be performed.

### Conclusions

The present study adds to the growing body of evidence that interpreting the results of next generation sequencing, particularly for 16 S rRNA diversity is not as straightforward as previously believed, and is riddled with potential biases. In general, polymerase affected both the diversity richness and community structure analysis; while template dilution and increasing the PCR cycle number reduced the richness, but did not affect community structure. Considering that the sequencing data from different environmental or human microbiome studies may be pooled together for comparing microbial diversity [24,25], these data should be interpreted carefully. We reiterate that samples should be performed on consistent PCR conditions for comparing microbial diversity, particularly for diversity richness.

## Methods

### DNA extraction

The sediment sample was taken from the Mai Po Ramsar wetland in Hong Kong, China. We collected a total of 250 g of four subsamples within 1 m diameter at the edge of the mangrove wetland, pooled them together, mixed them well, and then used 1 g for DNA extraction. The mangrove was vegetated with *Kadelia candel *and *Acanthus ilicifolius*. The sediment was collected in Aug 2009, and the DNA was extracted from the fresh sediment using the Ultraclean Soil DNA kit (MoBio, USA). The DNA was quantified using the NanoDrop and the concentration was 34 ng μl^-1^.

### PCR amplification

We used the 967F (CNACGCGAAGAACCTTANC) and 1046R (CGACAGCCATGCANCACCT) primers to amplify bacterial 16 S V6 fragments. An 8-digit error-correcting barcode sequence (Table 1) as described by Hamady et al. [26] was added before the 5' end of the 967F primer. A 2 bp 'GT' linker was added between the barcode and the 5' end of the 967f primer to avoid the potential match of barcode sequence with target 16 S sequences. The ultra PAGE purified primers were ordered from Sangon, China.

For each sample, one tube of PCR was performed. The PCR cycle condition was an initial denaturation at 94°C for 2 min; 25 or 30 cycles of 94°C 30 s, 57°C 30 S and 72°C 30S; and a final extention at 72°C for 5 min. The template dilution fold, the cycle number and the polymerase used were as listed in the table 1. For A, B, C, and D groups, each 20 μl reaction consisted of 2 μl Takara 10× Ex Taq Buffer (Mg^2+ ^plus), 2 μl dNTP Mix (2.5 mM each), 0.5 μl Takara *Ex Taq *DNA polymerase (2.5 units), 1 μl template DNA, 1 μl 10 μM barcoded primer 967F, 1 μl 10 μM primer 1406R, and 12.5 μl ddH_2_O. For condition E, each 20 μl reaction consisted of 10 μl *PfuUltra II *Hotstart 2× Master Mix, 1 μl template DNA, 1 μl 10 μM barcoded primer 967F, 1 μl 10 μM primer 1406R, and 7 μl ddH_2_O.

### Deep sequencing using Solexa GAII

Barcode tagged 16 S V6 PCR products were pooled, purified (QIAquick PCR purification Kit, Qiagen), end repaired, A-tailed and pair-end adaptor ligated (Pair-end library preparation kit, Illumina). After the ligation of the adaptors, the sample was purified and dissolved in 30 μl of elution buffer, and 1 μl was then used as template for 12 cycles of PCR amplification. The PCR product was gel purified (QIAquick gel extraction kit, Qiagen) and directly sequenced using the 75 bp pair-end strategy on the Solexa GA II following the manufacturer's instructions. The base-calling pipeline (version SolexaPipeline-0.3) was used to process the raw fluorescent images and the call sequences.

### Data analysis

The paired-end reads were overlapped to assemble the final sequence of V6 tags. The sequencing quality of the Solexa platform decreases near the 3' end. We used the first 60 bp from the 5' end of each read for overlapping assembly. A pair was connected with a minimum overlap length of 5 bp and 0 mismatches in the overlapped region. We further trimmed all tags with any mismatches within primers, with any N bases or less than 35 bp for the V6 regions. The final high quality tags were allocated to each sample according to the barcode sequence.

We performed taxonomic classification by assigning the reads of each sample to the 16 S V6 region database refhvr_V6 and then calculated the Global Alignment for Sequence Taxonomy (GAST) distance [27] (blastn release:2.2.18, e-value <1e-5, -b 50, http://vamps.mbl.edu/resources/databases.php). The OTU, rarefaction, Chao1 and ACE estimation were analyzed using the mothur (v.1.6.0, http://www.mothur.org/wiki/Main_Page) [18]. We wrote a Perl script to calculate the unique sequences (tags) and their abundance information for analyzing the rank-abundance curve of top abundant tags. The principal component analysis (PCA) was performed using Canoco (Version 4.51). The clustering analysis was performed using Primer 6.0. The sequences were deposited in NCBI Short Read Archive: SRA001401.

## Authors' contributions

JYW participated in the design of the study and performed the molecular experiments, XTJ participated in the bioinformatics analysis, SYL participated in the molecular experiments, FZ participated in the design of the study, HWZ participated in the design of the study, analyze the data and draft the manuscript. All authors read and approved the final manuscript.

## Supplementary Material

Additional file 1 **Rarefaction curves for unique and 0.03 OTU using the furthest, average and nearest neighbor clustering methods.** B1 and B2 samples had the same PCR condition but with different sequencing depth. A figure showing rarefaction curves of a couple of replicate samples calculated with different clustering methods. Click here for file

Additional file 2 **Rarefaction curves at 0.05 and 0.1 distances**. A figure showing rarefactions curves at 0.05 and 0.1 distances for samples as shown in the Fig. 1. Click here for file

Additional file 3 **Mass spectrum determination of the upstream barcoded primer 967F**. A figure showing the quality control of primer 967F using mass spectrum. Click here for file
