# Supplementary figures and images for: Effects of polymerase, template dilution and cycle number on PCR based 16 S rRNA diversity analysis using the deep sequencing method
Source: BMC Microbiol. 2010 Oct 12;10:255. doi: 10.1186/1471-2180-10-255 (PMC2964677; doi:10.1186/1471-2180-10-255)

## Slide 1
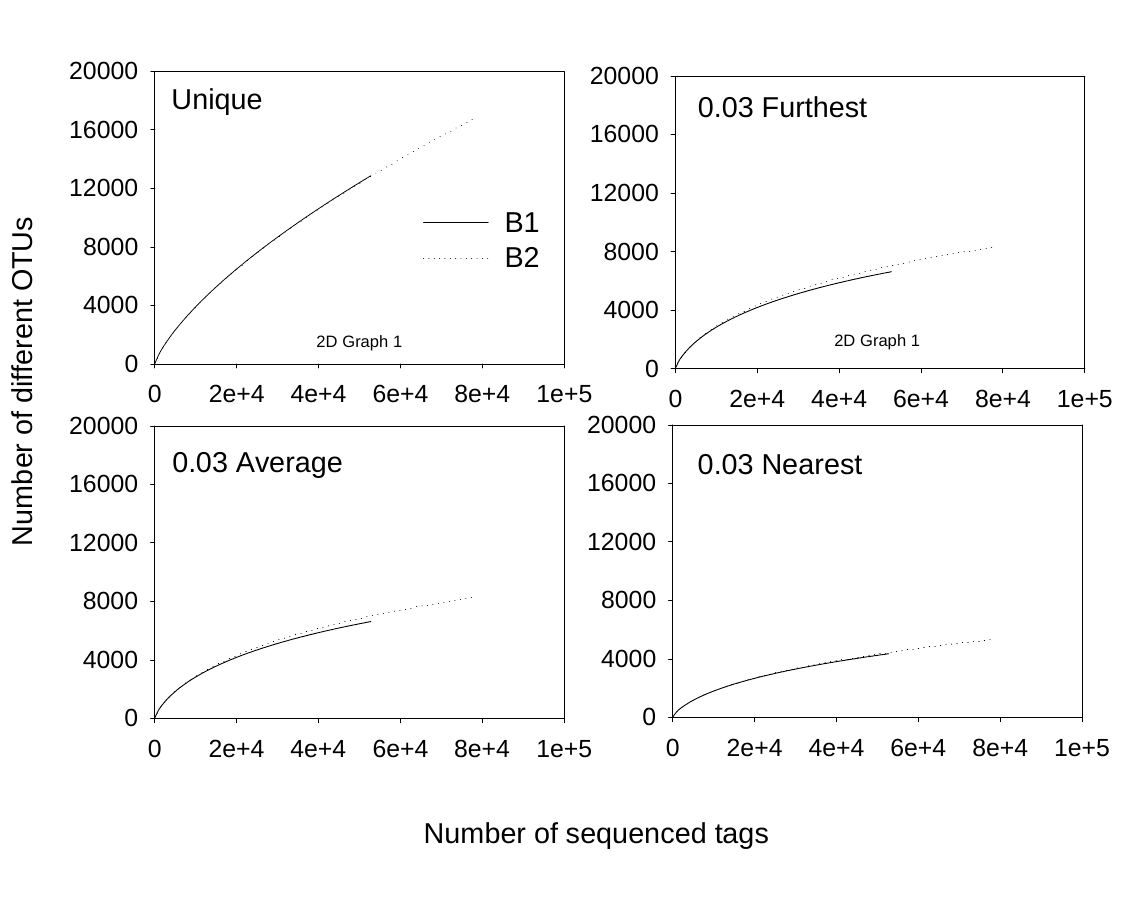

#

Supplement: Additional file 1 — Rarefaction curves for unique and 0.03 OTU using the furthest, average and nearest neighbor clustering methods. B1 and B2 samples had the same PCR condition but with different sequencing depth. A figure showing rarefaction curves of a couple of replicate samples calculated with different clustering methods. [file 1471-2180-10-255-S1.PPT]

## Slide 1
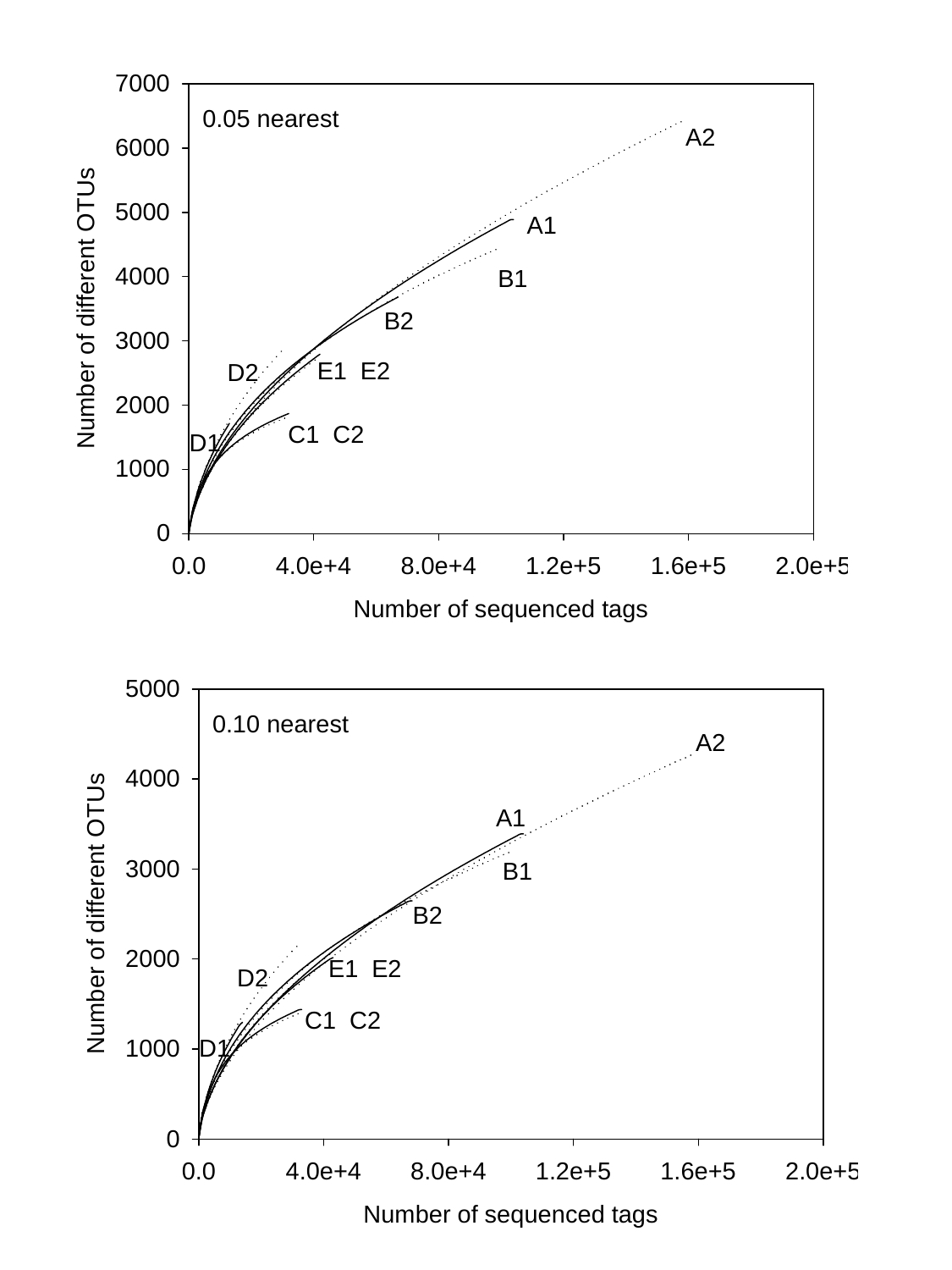

Supplement: Additional file 2 — Rarefaction curves at 0.05 and 0.1 distances. A figure showing rarefactions curves at 0.05 and 0.1 distances for samples as shown in the Fig. 1. [file 1471-2180-10-255-S2.PPT]

## Slide 1
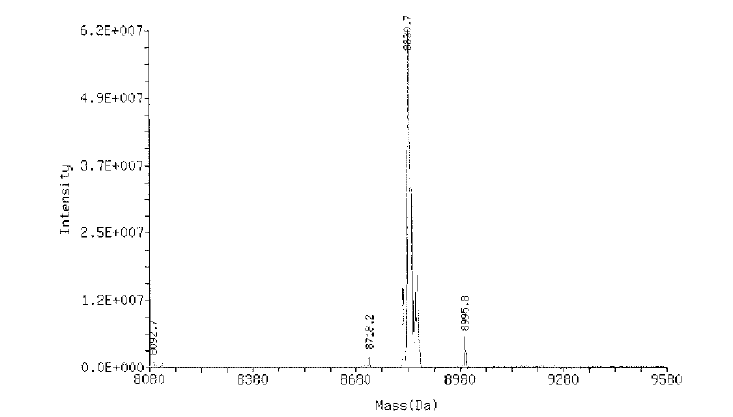

Supplement: Additional file 3 — Mass spectrum determination of the upstream barcoded primer 967F. A figure showing the quality control of primer 967F using mass spectrum. [file 1471-2180-10-255-S3.PPT]
